# Clinical and genetic characterization of leukoencephalopathies in adults

**DOI:** 10.1093/brain/awx045

**Published:** 2017-03-02

**Authors:** David S. Lynch, Anderson Rodrigues Brandão de Paiva, Wei Jia Zhang, Enrico Bugiardini, Fernando Freua, Leandro Tavares Lucato, Lucia Inês Macedo-Souza, Rahul Lakshmanan, Justin A. Kinsella, Aine Merwick, Alexander M. Rossor, Nin Bajaj, Brian Herron, Paul McMonagle, Patrick J. Morrison, Deborah Hughes, Alan Pittman, Matilde Laurà, Mary M Reilly, Jason D Warren, Catherine J Mummery, Jonathan M. Schott, Matthew Adams, Nick C. Fox, Elaine Murphy, Indran Davagnanam, Fernando Kok, Jeremy Chataway, Henry Houlden

**Affiliations:** 1 Department of Molecular Neuroscience, UCL Institute of Neurology, London, UK; 2 Leonard Wolfson Experimental Neurology Centre, UCL Institute of Neurology, London, UK; 3 Neurogenetics Unit, Neurology Department, Hospital das Clínicas da Universidade de São Paulo, São Paulo, Brazil; 4 MRC Centre for Neuromuscular Diseases, UCL Institute of Neurology, London, UK; 5 Instituto de Radiologia, Hospital das Clínicas da Universidade de São Paulo, São Paulo, Brazil; 6 Centro de Estudos do Genoma Humano, Universidade de São Paulo, São Paulo, Brazil; 7 Lysholm Department of Neuroradiology, National Hospital for Neurology and Neurosurgery, Queen Square, London, UK; 8 Neurology Department, St. Vincent’s University Hospital and University College Dublin, Ireland; 9 Charles Dent Metabolic Unit, National Hospital for Neurology and Neurosurgery, Queen Square, London, UK; 10 Chelsea and Westminster NHS Foundation Trust, London, UK; 11 Sobell Department of Motor Neuroscience and Movement Disorders, UCL Institute of Neurology, London, UK; 12 Department of Neurology, Queens Medical Centre, Nottingham, UK; 13 Department of Neuropathology, Royal Victoria Hospital, Belfast, Northern Ireland, UK; 14 Department of Neurology, Royal Victoria Hospital, Belfast, Northern Ireland, UK; 15 Centre for Cancer Research and Cell Biology, Queens University of Belfast, 97 Lisburn Road, Belfast BT9 7AE, UK; 16 Dementia Research Centre, UCL Institute of Neurology, London, UK; 17 Department of Neuroinflammation, UCL Institute of Neurology, London, UK; 18 Neurogenetics Laboratory, National Hospital for Neurology and Neurosurgery, Queen Square, London, UK

**Keywords:** leukodystrophy, neurodegeneration, white matter lesion, imaging

## Abstract

Leukodystrophies and genetic leukoencephalopathies are a rare group of disorders leading to progressive degeneration of cerebral white matter. They are associated with a spectrum of clinical phenotypes dominated by dementia, psychiatric changes, movement disorders and upper motor neuron signs. Mutations in at least 60 genes can lead to leukoencephalopathy with often overlapping clinical and radiological presentations. For these reasons, patients with genetic leukoencephalopathies often endure a long diagnostic odyssey before receiving a definitive diagnosis or may receive no diagnosis at all. In this study, we used focused and whole exome sequencing to evaluate a cohort of undiagnosed adult patients referred to a specialist leukoencephalopathy service. In total, 100 patients were evaluated using focused exome sequencing of 6100 genes. We detected pathogenic or likely pathogenic variants in 26 cases. The most frequently mutated genes were *NOTCH3*, *EIF2B5*, *AARS2* and *CSF1R.* We then carried out whole exome sequencing on the remaining negative cases including four family trios, but could not identify any further potentially disease-causing mutations, confirming the equivalence of focused and whole exome sequencing in the diagnosis of genetic leukoencephalopathies. Here we provide an overview of the clinical and genetic features of these disorders in adults.

## Teaser

Leukoencephalopathies are a diverse group of white matter disorders that can be difficult to diagnose. Using focused and whole-exome sequencing, Lynch *et al*. expand the known clinical and mutational spectrum of genetic leukoencephalopathy in adulthood, and describe the frequency and clinical and radiological phenotype of the most commonly mutated genes.

## Introduction

Leukodystrophies and genetic leukoencephalopathies represent a diverse group of disorders in which there is progressive degeneration of CNS white matter. They can present in children or adults, often with a constellation of clinical features including dementia, movement disorders, ataxia and upper motor neuron signs, accompanied by hyperintense signal abnormalities in the brain/spinal cord on T_2_-weighted MRI (Vanderver *et al.*, 2015).

There are at least 60 genes implicated in the development of these disorders, involved in diverse cellular pathways including myelin formation, mitochondrial health and protein translation (Parikh *et al.*, 2015). This heterogeneity, in combination with often overlapping clinical and radiological phenotypes, make definitive diagnosis challenging. In addition, the large number of genes and pathways involved means that the traditional approach to diagnosis, involving extensive metabolic and biochemical testing, is time consuming, costly, and often fails to reach a diagnosis (Richards *et al.*, 2015*a*).

In recent years, advances have been made in the understanding of leukodystrophy and leukoencephalopathy in children, with particular emphasis on improvements in diagnostic approach (Vanderver *et al.*, 2016). However, little is known about the genetic spectrum of these disorders in adults, where in many cases a definitive diagnosis is not reached.

In this study, we used focused and whole exome sequencing (WES) to genetically evaluate a cohort of 100 adult patients with undiagnosed leukodystrophy/leukoencephalopathy, describe the clinical spectrum of these disorders in adults and explore the efficiency of next generation sequencing in their diagnosis.

## Materials and methods

### Patient recruitment

Patients were recruited from the Queen Square Adult Leukodystrophy Group (QSALG), a multidisciplinary expert group that accepts referrals on patients with undiagnosed leukodystrophy/leukoencephalopathy, the Dementia Research Centre and Neurogenetics Departments of the National Hospital for Neurology and Neurosurgery, London and the Neurology Department of the University of São Paulo, Brazil. The primary inclusion criteria included the presence of a progressive neurological syndrome with prominent T_2_-weighted hyperintensity of the cerebral/spinal cord white matter, which was confluent and not consistent with an acquired inflammatory cause such as multiple sclerosis. Haematological, biochemical and metabolic testing was performed by the referring centre according to published guidelines (Ahmed *et al.*, 2014). All patients underwent Round 1 investigations to exclude acquired causes such as infections or autoimmune syndromes, and Round 2 investigations to exclude the classical leukodystrophies (white cell enzyme studies, very long chain fatty acids, amino/organic acids and bile alcohols). Some patients also underwent additional muscle/nerve or brain biopsy. Patients were only included if a diagnosis could not be reached after these investigations. Informed consent was obtained from all study participants for genetic analysis and the study was carried out with institutional ethical approval. All patients were at least 16 years old at the time of referral to our centres.

### Focused exome and whole exome sequencing

Genomic DNA was extracted from peripheral blood lymphocytes. Focused exome sequencing was performed using the Agilent Sure Select Focused Exome (Agilent) according to the manufacturer’s protocol. WES was performed using Nextera chemistry (Illumina Inc). Sequencing was performed using the HiSeq platform (Illumina).

The Illumina fastq sequencing data were mapped to the human reference assembly, hg19 (GRCh37; UCSC genome browser) by Novoalign Software (Novocraft Inc). After removal of PCR duplicates (Picard) and reads without a unique mapping location, variants were extracted using the Maq model in SAMtools and outputted by the following criteria: consensus quality > 30, SNP quality > 30 and root mean square mapping quality > 30. These variant calls were then annotated using Annovar software (Wang *et al.*, 2010).

### Variant prioritization

We initially prioritized variants in known leukodystrophy/genetic leukoencephalopathy genes as recommended by the GLIA consortium in 2015 (Parikh *et al.*, 2015). We removed synonymous/intronic variants and variants with minor allele frequency (MAF) > 0.01 on the ExAC database.

Potentially pathogenic variants were confirmed using conventional Sanger sequencing and all variants were classified using American College of Medical Genetics (ACMG) criteria (Richards *et al.*, 2015*b*).

## Results

**Figure 1:**
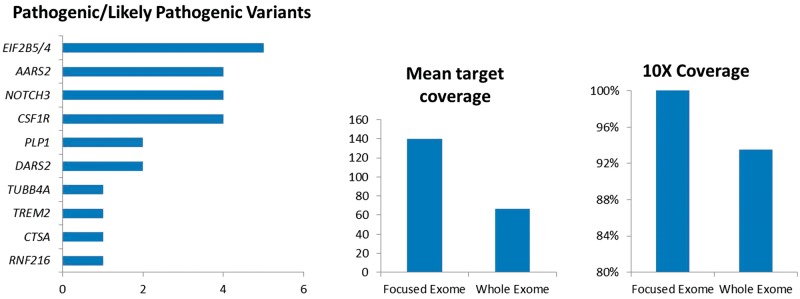
**Pathogenic and likely pathogenic variants identified in known leukodystrophy/genetic leukoencephalopathy genes and coverage metrics for focused exome and WES**.

Focused exome sequencing identified pathogenic or likely pathogenic variants in known leukodystrophy/leukoencephalopathy genes in 26 cases (Table 1). The most frequently mutated genes were *EIF2B4*/*5* causing vanishing white matter (VWM), *CSF1R*, causing adult onset leukoencephalopathy with axonal spheroids and pigmented glia (ALSP), *AARS2*, causing a leukoencephalopathy with ovarian failure and *NOTCH3*, causing cerebral autosomal dominant arteriopathy with subcortical infarcts and leukoencephalopathy (CADASIL) (Fig. 1). We detected variants of uncertain significance in *POLR3A*, *MRPS22* and *TNR* in three patients (Supplementary material).

**Table 1:** Pathogenic and likely pathogenic variants detected in known leukodystrophy and leukoencephalopathy genes

Patient ID	Gene	Variant	Predicted protein effect	Sex	AAO	Family history	Initial syndrome	Additional features
**Vascular leukoencephalopathy**								
P1	*NOTCH3*	c.505C > T	p.R169C	M	30	AD	Migraine with aura	Parietal ischaemic stroke
P2	*NOTCH3*	c.994C > T	p.R332C	M	37	AD	Recurrent TIA	Progressive gait deterioration, dysarthria, cognitive decline
P3	*NOTCH3*	c.397C > T	p.R133C	F	36	No	Migraine with aura	Ischaemic stroke, cognitive decline
P4	*NOTCH3*	c.1591T > G	p.C531G	F	36	AD	Prepontine SAH	Migraine with aura
P28	*CTSA*	c.973C > T	p.R325C	F	42	AD	Migraine, hypertension	Mild cognitive decline, behavioural change
**Vanishing white matter disease**								
P5	*EIF2B4*	c.[495 + 3delA (;) 623G > A]	p.[? (;) R208Q]	F	20	AR	Epilepsy	Spastic tetraparesis, ataxia, cognitive decline
P6	*EIF2B5*	c.[338G > A];[380T > C]	p.[R113H];[L127P]	F	53	No	Cognitive decline	Migraine, ataxia, personality change
P7	*EIF2B5*	c.[338G > A];[913A > T]	p.[R113H];[M305L]	F	52	No	Trigeminal neuralgia	Temporal seizures, ataxia, cognitive decline
P8	*EIF2B5*	c.[338G > A];[338G > A]	p.[R113H];[R113H]	M	31	No	Slowly progressive hemiparesis	Cognitive decline, ataxia
P26	*EIF2B5*	c.[338G > A];[338G > A]	p.[R113H];[R113H]	M	26	No	Cognitive decline	Falls, parkinsonism
**Hypomyelinating disorders**								
P9	*PLP1*	c.276C > A	p.Y92*	F	30	AD/XLD	Spastic gait, ataxia	Executive dysfunction, impaired visual memory
P10	*PLP1*	c.355dupG	p.Q121Pfs*83	M	30	No	Spastic gait, head tremor,	Executive dysfunction, cognitive decline, bladder/bowel disturbance
P12	*TUBB4A*	c.G538G > A	p.V180M	F	Infancy	*De novo*	Spasticity, dystonia	Dysarthria, cognitive decline
**Mitochondrial proteins**								
P13	*DARS2*	c.[228‐20_-21 delTTinsC];[1433T > C]	p.[R76Sfs*5];[F479S]	M	15	No	LL weakness, neuropathy	Ataxia
P14	*DARS2*	c.[228‐20_-21 delTTinsC];[1456C > T]	p.[R76Sfs*5];[L486F]	F	5	No	Bilateral UL intention tremor, progressive gait impairment	Cognitive decline, spastic paraplegia, sensory ataxia
**Genes involved in microglial signalling**								
P16	*CSF1R*	c.1901T > G	p.L634R	F	53	No	Ataxia, spasticity	Cognitive, dyspraxia
P17	*CSF1R*	c.2570C > T	p.P857L	F	38	No	Spastic gait	Severe spastic tetraparesis, dystonia, cognitive decline
P18	*CSF1R*	c.2381T > C	p.I794T	M	52	No	Cognitive decline	UL dyspraxia, alien limb, spasticity, parkinsonism
P27	*CSF1R*	c.2345G > A	p.R782H	M	46	AD	Cognitive decline	Depression, apraxia
P19	*TREM2*	c.[377T > G];[377T > G]	p.[V126G];[V126G]	M	25	AR	Cognitive decline	Frequent generalized seizures
**Miscellaneous**								
P21	*RNF216*	c.[1482C > A];[1482C > A]	p.[Y494*];[Y494*]	M	31	Consang	Cognitive decline	Ataxia, hypogonadotrophic hypogonadism

AAO = age at onset; AD = autosomal dominant; AR = autosomal recessive; XLD = X-linked dominant; consang = consanguineous relationship; SAH = subarachnoid haemorrhage; TIA = transient ischaemic attack; UL = upper limb; LL = lower limb.

To assess the utility of focused exome sequencing compared to WES we sequenced the remaining 71 negative cases (including four family trios) using WES. However, we did not make any further diagnoses from this approach, confirming that in adult leukoencephalopathies, WES offers no benefit over focused exome sequencing. This is likely because the target coverage is better on focused exome compared to WES. In our study, coverage metrics showed that focused exome had mean target coverage of 140 with 99% of targeted bases read > 10 times, compared to WES where mean targeted coverage was 66 with 94% of targeted bases read >10 times (Fig. 1).

It was not possible to predict prospectively which cases were likely to be positive for a known disease gene. While negative cases had a slightly older age of onset (mean 35 years) compared to positive cases (mean 32 years), this was not statistically different (unpaired *t*-test *P-*value 0.50). Similarly, while more positive cases had a family history (positive cases 42%, negative cases 24%), this was also not statistically significant (Fisher’s exact test *P-*value 0.10). There was no difference in typical clinical features or MRI appearance between the two groups (Supplementary Table 1).

### Disease spectrum

#### Vanishing white matter

We identified five patients with VWM disease, four with mutations in *EIF2B5* and one with mutations in *EIF2B4.* The average age of onset of neurological symptoms was 36 years. The most frequent symptoms reported were cognitive decline, ataxia and epilepsy. Of the three female cases, two experienced premature ovarian failure, with the remaining female case undergoing menopause at age 45 years. One patient (Patient P7) presented with trigeminal neuralgia aged 52 years, and was initially diagnosed with multiple sclerosis following an abnormal MRI scan. Ten years later, she developed partial epilepsy and cognitive decline and a repeat MRI showed findings typical of VWM. In all cases, MRI demonstrated bilateral confluent T_2_-weighted/FLAIR (fluid-attenuated inversion recovery) hyperintensities with white matter rarefaction and global atrophy.

#### CADASIL

**Figure 2:**
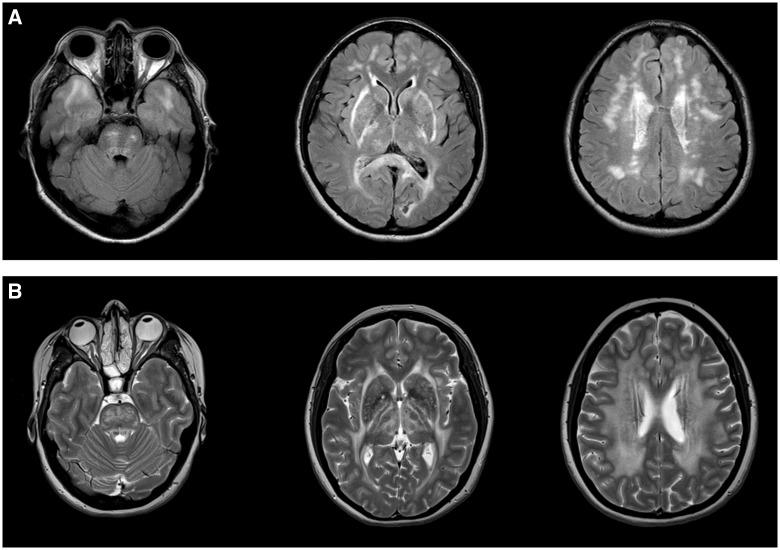
**CADASIL and CARASAL imaging appearance. **(**A**) Typical imaging appearance of CADASIL in axial FLAIR MRI images. There is symmetric subcortical high signal in the anterior temporal lobes, internal and external capsules and scattered asymmetric involvement of the periventricular cerebral white matter and pons. (**B**) CARASAL in T_2_ axial images. There was no involvement of the anterior temporal lobes (*left*) but there was extensive involvement of the internal and external capsules, the basal ganglia and thalami (*middle*) and the periventricular and deep white matter (*right*).

We identified four patients with mutations in the *NOTCH3* gene. The average age at onset for these patients was 35 years. Ischaemic stroke and migraine with aura were the most common symptoms, occurring in three of four patients. One patient (Patient P4) presented with a prepontine subarachnoid haemorrhage. Cognitive decline was mild in all cases. One patient (Patient P4) had no progression 5 years from symptom onset; the remaining three patients had mild cognitive impairment only after 10 years of follow-up. The *NOTCH3* cases were the only patients to demonstrate T_2_-weighted/FLAIR hyperintensity in the temporal poles (Fig. 2A), although this was not universal, with Patient P1 not showing temporal pole abnormalities on imaging taken more than 10 years after symptom onset.

#### CARASAL

We identified a single patient with the recently described syndrome of cathepsin A-related arteriopathy with strokes and leukoencephalopathy (CARASAL) (Bugiani *et al.*, 2016). The patient had a history of migraine since age 20 in addition to hypertension and depression. She developed persistent facial pain at age 42 years, which was accompanied by very mild cognitive symptoms consisting of problems with episodic memory and facial recognition. By age 48, the syndrome had progressed and the patient developed mild behavioural changes with some disinhibition. Mini-Mental State Examination was 27/30 and performance IQ was 99. The neurological examination was normal. The patient’s father had died from stroke at age 60 years but no other family members were known to be affected. MRI demonstrated extensive signal abnormality in the deep and periventricular white matter sparing the U-fibres, extending into the thalamus, basal ganglia, pyramidal and tegmental tracts, the superior and middle cerebellar peduncles and the right dentate nucleus. A few old lacunes were demonstrated in the deep grey matter nuclei, although no acute infarcts or microhaemorrhages were present (Fig. 2B). Both the history and imaging appearance strongly suggested CADASIL, but no *NOTCH3* mutation was identified. The same heterozygous *CTSA* mutation, c.973C > T, p.R325C, described in two families by Bugiani *et al.* (2016) was detected by focused exome sequencing, confirming the diagnosis of the novel entity CARASAL.

#### CSF1R

We identified four patients with mutations in *CSF1R,* which causes ALSP (Rademakers *et al.*, 2011). Mean age of onset was 47 years. Two patients (Patients P22 and P27) presented with cognitive decline and psychiatric symptoms followed later by upper limb dyspraxia, alien limb phenomenon and parkinsonism. Patients P20 and P21 presented with ataxia and spasticity, and later developed cognitive decline. In all cases, there was significant cognitive decline within 2 years of onset. MRI in all cases demonstrated slightly asymmetric T_2_ hyperintense signal abnormality in the cerebral white matter affecting the frontal, parietal and temporal lobes with frequent involvement of the corticospinal tracts and atrophy of the cerebral white matter and corpus callosum. Punctate areas of restricted diffusion were also evident in the cerebral white matter, which persisted and sometimes increased over time.

#### AARS2

**Figure 3:**
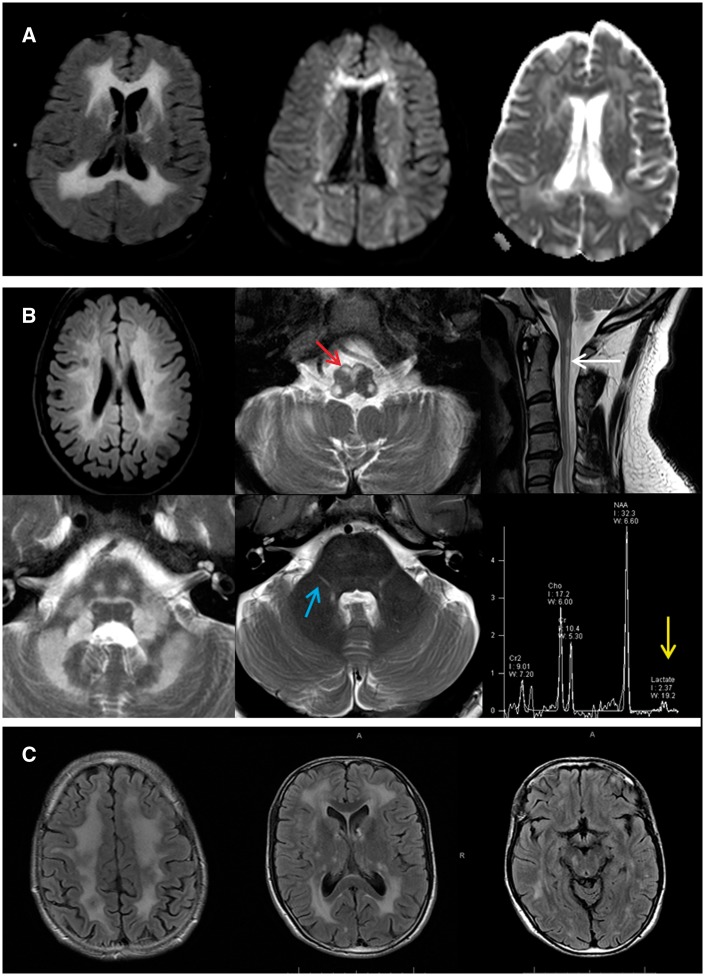
***AARS2, DARS2* and *RNF216* imaging appearance. **(**A**) Typical imaging appearance of AARS2-related leukoencephalopathy. There is an extensive, symmetric white matter abnormality in the frontal, parietal and occipital lobes on the FLAIR imaging (*left*), with evidence of restricted diffusion on diffusion weighted imaging (*middle*) and apparent diffusion coefficient (*right*). (**B**) DARS2-related leukoencephalopathy. Axial FLAIR MRI image (*top left*) discloses bilateral deep and periventricular cerebral white matter involvement. Axial and sagittal T_2_-weighted MRI images show hyperintensity in the medullary pyramids (red arrows), the posterior columns of the spinal cord (white arrow), the deep white matter of the cerebellum and along the intraparenchymal tracts of the trigeminal nerves bilaterally (blue arrows), in addition to a lactate peak on short echo time magnetic resonance spectroscopy (yellow arrow). (**C**) RNF216-related leukoencephalopathy with diffuse supratentorial white matter signal abnormality appreciated in axial FLAIR MRI images.

Five patients were identified with biallelic mutations in *AARS2*, which has recently been associated with a novel ovario-leukodystrophy (Dallabona *et al.*, 2014). The clinical details and mutations detected are reported in more detail elsewhere (Lynch *et al.*, 2016). Briefly, the phenotype consisted of adult onset leukoencephalopathy with a variable combination of dementia, upper motor neuron signs, ataxia and ovarian failure in females. MRI revealed slightly asymmetric abnormal T_2_ hyperintense signal in the frontoparietal and periventricular white matter, with white matter rarefaction, involvement of the corpus callosum and pyramidal tracts and punctate areas of restricted diffusion, reminiscent of ALSP (Fig. 3A). In one case for which brain tissue was available, frequent axonal spheroids and pigmented microglia were seen.

#### Hypomyelinating disorders

We identified three patients with mutations in genes causing hypomyelination. Two patients were found to carry loss-of-function mutations in *PLP1.* Both patients developed symptoms at age 30 years. A female patient (Patient P9), carrying a novel nonsense mutation, presented with progressive spastic paraplegia and gait ataxia, followed by executive dysfunction and impaired visual memory. It has been shown that symptoms manifest more commonly in female patients carrying nonsense mutations in *PLP1* (Hurst *et al.*, 2006). A male patient (Patient P10), carrying a novel frameshift mutation, presented with a ‘no-no’ head tremor, gait disturbance and bowel and bladder symptoms at age 30 years. On examination at age 40 years, there was severe cognitive impairment with frontal release signs, severe spastic paraparesis requiring wheelchair use, ideomotor apraxia and near constant head tremor. Both patients demonstrated mildly increased cerebral white matter signal on T_2_-weighted/FLAIR imaging, with the white matter appearing isointense on T_1_-weighted images, indicating hypomyelination.

We identified a novel *de novo* mutation in *TUBB4A* in a 24-year-old female with a childhood onset dystonic syndrome with severe lower limb spasticity, dysarthria, cognitive decline and epilepsy (Patient P12). MRI revealed extensive T_2_-weighted hyperintensity of the periventricular and cerebellar white matter extending to the posterior limb of the internal capsules. The white matter appeared normal on T_1_-weighted imaging. The basal ganglia and cerebellum were not visibly involved by age 24 years.

#### DARS2

We detected two patients with novel compound heterozygous mutations in *DARS2*, the mitochondrial aminoacyl tRNA synthetase for aspartic acid. Recessive mutations in this gene are associated with the disorder leukoencephalopathy with brainstem and spinal cord involvement and lactate elevation (LBSL) (Scheper *et al.*, 2007). Patient P17 presented at age 8 years with exercise-induced calf cramps. By age 16, he had developed distal weakness and wasting with impaired deep tendon reflexes and pes cavus. By age 23 there was bilateral extensor plantars and he was diagnosed with hereditary spastic paraplegia with distal amyotrophy. The patient continued to decline and by his 40s had developed a moderate gait ataxia with severe distal weakness and sensory neuropathy. MRI revealed T_2_ hyperintense signal abnormality in the cerebral white matter, splenium of the corpus callosum, medullary pyramids, medial lemnisci, intraparenchymal trigeminal nerves, superior, middle and inferior cerebellar peduncles, cerebellar white matter and dorsal columns and lateral corticospinal tracts in the spinal cord. A lactate peak was detected on intermediate and long echo time magnetic resonance spectroscopy. These findings met the radiological criteria for diagnosis of LBSL, and the diagnosis was confirmed by the detection of the compound heterozygous *DARS2* variants. Patient P18 developed upper limb intention tremor and slowly progressive gait impairment at age 5. There was slow progression and gradual development of a sensory ataxia. By age 25 she had spastic paraplegia with reduced verbal fluency and mild cognitive impairment. The MRI was consistent with LBSL and this was confirmed by the presence of compound heterozygous *DARS2* mutations (Fig. 3B).

#### RNF216

We identified a novel homozygous nonsense mutation in *RNF216* in a 42-year-old male with slowly progressive cognitive decline, gait impairment and erectile dysfunction, starting at age 31 years. A brother was also affected, with a more rapid progression and death at age 38 years. On examination, there was evidence of mild cognitive impairment (MMSE 25/30), mild cerebellar ataxia and choreic movements of the hands and face. MRI demonstrated a symmetrical and diffuse supra and infra-tentorial leukoencephalopathy with involvement of the thalamus, external capsules, pons, middle cerebellar peduncles and dentate nucleus (Fig. 3C).

## Discussion

We used focused exome sequencing as an approach to diagnosis in a heterogeneous group of unsolved adult leukodystrophies/genetic leukoencephalopathies. All of the patients in our cohort had previously been extensively investigated without diagnosis. In a cohort of 100 patients, we made a definitive diagnosis in 26. This diagnostic rate compares favourably to other studies using a similar approach. In particular, Vanderver *et al.* (2016) made a diagnosis in 25 cases from 71 families, all of whom were childhood onset cases with full family trios. In general, diagnostic rates in adult onset genetic disorders tend to be lower than in children. This is because the population is more heterogeneous, often containing some patients with acquired disorders such as severe small vessel disease and because familial DNA is not often available in adult onset cases.

In some leukodystrophy/genetic leukoencephalopathy syndromes there is a close phenotype–genotype relationship allowing for accurate pre-test prediction. For example, the presence of ataxia and hypogonadotrophic hypogonadism with leukoencephalopathy strongly suggests Gordon Holmes syndrome due to *RNF216* mutations. Similarly, particular MRI patterns might significantly reduce the number of genes to screen, such as when hypomyelination is the predominant feature or the specific and striking features of LBSL. However, there remain a large number of patients who all present in a similar way with a subacute dementing illness with pyramidal and extrapyramidal features and a symmetric, non-specific white matter abnormality. In these cases, extensive biochemical or metabolic testing is less likely to be helpful after initial screening is performed (Ahmed *et al.*, 2014). Without specific pretest probability, sequential candidate gene sequencing is time-consuming, expensive and can significantly delay diagnosis. By contrast focused exome sequencing is an efficient approach to diagnosis as it simultaneously covers all known genes causing leukodystrophies/genetic leukoencephalopathies.

The number of new genes implicated in genetic leukoencephalopathies is growing each year. This provides challenges for the use of targeted panels, which inevitably become obsolete when a number of new genes are described. Using a focused exome approach offsets this, as the data can be reanalysed intermittently in light of new genetic discoveries and compares favourably with regard to coverage when compared to WES.

In summary, focused exome sequence performs as well as WES in the diagnosis of adult onset leukodystrophies, providing a definite genetic diagnosis in 26%, and revealing variants of uncertain significance in 3%. The 71 negative cases in our cohort continue to present a diagnostic challenge. Some of these may not have a Mendelian monogenic disorders, being either acquired, or due to the interaction of numerous genetic variants in a polygenic fashion. Some patients may carry pathogenic intronic variants in known genes or other mutations not readily detected by NGS including heterozygous copy number variants. Finally, some patients will carry pathogenic variants in genes not yet linked to leukodystrophies/genetic leukoencephalopathies. Further whole exome/genome sequencing along with family segregation and functional studies may in future resolve some, if not all, of these cases.

## Supplementary Material

Supplementary Data

## Abbreviation

WES: whole exome sequencing
