# Supplementary material for: Clinical and genetic characterization of leukoencephalopathies in adults
Source: Brain. 2017 Mar 2;140(5):1204–11. doi: 10.1093/brain/awx045 (PMC5405235; doi:10.1093/brain/awx045)
Supplement: Supplementary Data [file awx045_Supp.zip › brain-2016-01749-File006.pdf]

Supplementary Information

Clinical and genetic characterisation of leukoencephalopathies in adults

Variants of uncertain significance

We identified three patients carrying variants of uncertain significance (VUS) which are discussed in detail below.

POLR3A

We identified novel, compound heterozygous *POLR3A* mutations (c.[2438T>A];[3718G>A]) in a 47 year old man with a 2 year history of asymmetric, non-Levodopa responsive Parkinsonism (P11). In the left arm, there was reduced arm swing, rest tremor and cogwheel rigidity. There was no progression over 4 years. MRI imaging was consistent with diffuse hypomyelination (Supplementary Figure 1). Familial studies confirmed that the *POLR3A* mutations were *in trans*, and the proband was the only member of his family to carry both mutations.

While the variants are rare on publically available databases, are conserved residues, and are *in trans*, they did not meet the strict ACMG criteria to be described as pathogenic/likely pathogenic and we have classed them as variants of uncertain significance.

Interestingly, there were no abnormalities of the teeth or features of hypogonadotrophic hypogonadism, which are often associated with *POLR3A* mutations in the '4H syndrome'. While almost all patients described with *POLR3* related leukodystrophy have been children, recently 2 adult siblings were described with mutations in a related gene, *POLR1C* (Geschwind et al, AAN, 2016).

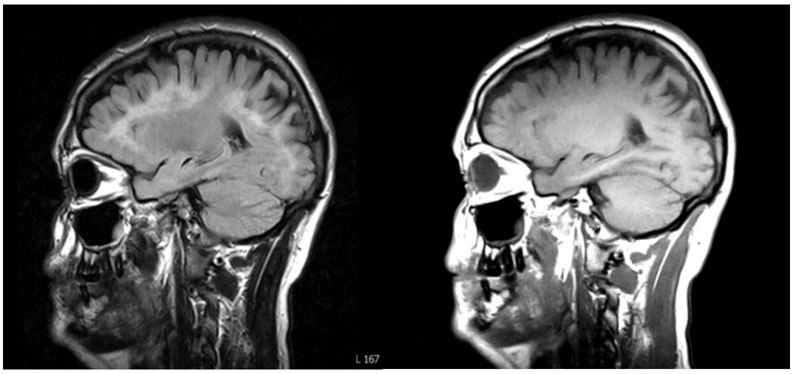

Supplementary Figure 1. Parasagittal FLAIR (left) and T1 (right) images are consistent with diffuse hypomyelination.

Species		Alignment	
Human		813 Q A I S G S R V P D G F E N R S L	1240 M A T H G V K G T R
mutated	all conserved	813 Q A I S G S R V P D G Y E N R S L	1240 M A T H **S** V K G T R
Ptroglodytes	all identical	770 Q A I S G S R V P D G F E N R S L	1197 M A T H G V K G T R
Mmulatta	all identical	813 Q A I S G S R V P D G F E N R S L	1240 M A T H G V K G T R
Fcatus	all identical	813 Q A I S G S R V P D G F E N R S L	1240 M A T H G V K G T R
Mmusculus	all identical	813 Q A I S G S R V P D G F E N R S L	1240 M A T H G V K G T R
Ggallus	all identical	813 Q A I S G S R V P D G F E N R S L	1240 M A T H G V K G T K
Trubripes	all identical	813 Q A I S G S R V P D G F E N R S L	1240 M A T H G V N G S R
Drerio	all identical	813 Q A I S G S R V P D G F E N R S L	1240 M A T H G V N G S K
Dmelanogaster	all identical	811 Q A I S G K R V P N G F E N R A L	1233 I A T Y G V V G K R
Celegans	all identical	809 Q A I S G H R P P D G F E E R S L	1254 L S S V G V D P R K

Conservation of c.2438T>A, p.F813Y and c.3718G>A, p.G1240S in *POLR3A*

TNR

We identified a novel homozygous frameshifting mutation in the *TNR* gene (c. 1475delG, p. R492Pfs*45), encoding an extracellular matrix protein expressed in the CNS. The patient (P24) was a 19 year old woman of Turkish origin. She was born of a consanguineous marriage and was normal from birth to the age of 3 months. She developed a floppy head and opisthotonic spasms of her neck and back. Her motor milestones were delayed, crawling at 1 year, and never standing or walking independently. By the age of 2 she had developed generalised dystonia and spasticity. Lower limb spasms occurred at age 4, resolved, and returned at age 19. Examination revealed a mixed spastic and cerebellar dysarthria. Saccades were slowed and interrupted. There was wasting and distal weakness in the upper limbs and spasticity and dystonic posturing. There was mild learning disability.

An MRI obtained at age 19 years revealed a large number of T2/FLAIR hyperintense lesions in subcortical and periventricular white matter, brainstem and cerebellum consistent with demyelination. Interval imaging demonstrated that new lesions had appeared within a 2 month

period (Supplementary Figure 2). Oligoclonal bands were found in the CSF. A presumptive diagnosis of Multiple Sclerosis was made and interferon treatment commenced.

This phenotype is strikingly similar to a child reported by Dufresne *et al* in 2012 where a Lebanese child of consanguineous parents developed opisthotonic spasms at 6 weeks(Dufresne *et al.*, 2012). This resolved, followed by choreoathetoid movements and the development of spastic quadriparesis and hyperreflexia. This child was found to carry a homozygous deletion on Chromosome 1q25.1, containing the *TNR* gene. The *TNR* gene is classified by ExAC as extremely intolerant of loss of function mutations with a pLi of 0.99 where a score >0.9 indicates genes which are extremely intolerant(ExAC, n.d.). This strongly supports the pathogenicity of this mutation in the origin of the childhood onset dystonic syndrome. However, the extent to which this contributes to the inflammatory disorder, which is consistent with MS, is unknown. *TNR* plays an important role in brain development and myelin formation, and is highly expressed in oligodendrocytes and oligodendrocyte precursors. When myelination is complete, expression of *TNR* reduces(Fuss *et al.*, 1993; Wintergerst *et al.*, 1993). This is the first adult described with a *TNR* loss of function mutation and whether this is associated with increased MS risk warrants further study.

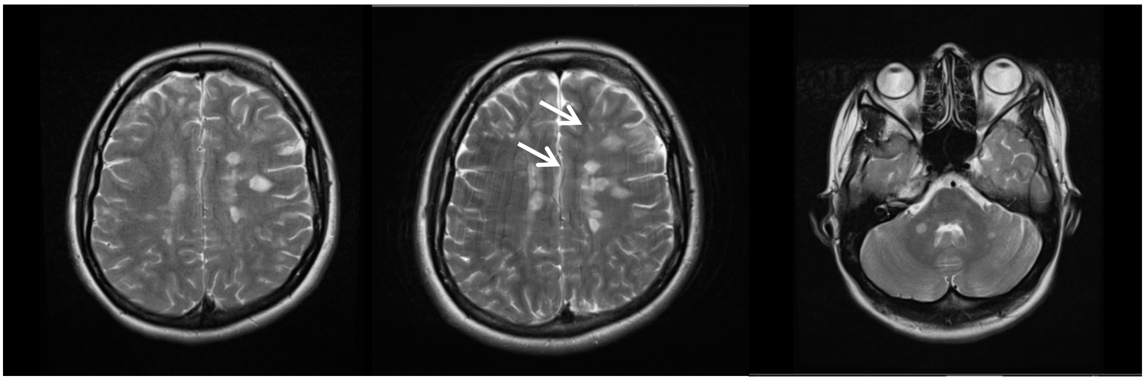

Supplementary Figure 2. Imaging appearance of P20 (axial T2-weighted MRI images) with a homozygous frameshift mutation in TNR showing new, demyelinating lesions in a typical inflammatory distribution in the supra- and infra-tentorial white matter (arrows).

MRPS22

We identified a single patient with a homozygous mutation in the mitochondrial ribosomal protein *MRPS22* (c. 605G>A, p. R202H) (P19). This patient is of Turkish origin and was born to consanguineous parents. His brother was also affected. Symptoms first developed age 30 with progressive cognitive impairment. This was followed later by the development of cerebellar ataxia and mixed myopathic and neuropathic signs. An MRI showed a symmetric leukoencephalopathy involving the frontal, parietal and occipital white matter, the posterior limb of the internal capsules and the dorsal pons and midbrain. The cerebellar white matter was also involved. A nerve biopsy demonstrated a mild axonal neuropathy affecting small myelinated and unmyelinated axons. There were 2 cytochrome oxidase negative fibres on muscle biopsy but the muscle was otherwise normal and respiratory chain enzyme analysis was normal. Cortical brain biopsy revealed only gliosis with no abnormal protein accumulation or demyelination.

We identified a novel homozygous mutation in the *MRPS22* gene. This residue is highly conserved amongst species and was heterozygous in an unaffected sibling. Previously mutations in *MRPS22* have been demonstrated in severe childhood onset mitochondrial disease with combined oxidative phosphorylation deficiency and defects in mitochondrial translation(Saada *et al.*, 2007; Smits *et al.*, 2011). More recently, 2 Tunisian siblings of consanguineous marriage were described with a childhood onset multisystem disorder with cerebral and cerebellar leukoencephalopathy with cystic changes, recurrent lactic acidosis, cardiac abnormalities and optic atrophy. These siblings carried a frameshift and missense mutation. In other disorders with defects in mitochondrial translation, e.g. *AARS2,* some mutations lead to fatal infantile disorders, while others lead to adult onset leukoencephalopathy, depending on the functional outcome of the particular variant. It is possible that the R202H variant is less disruptive to protein function than the previously described R170H or L215P mutations.

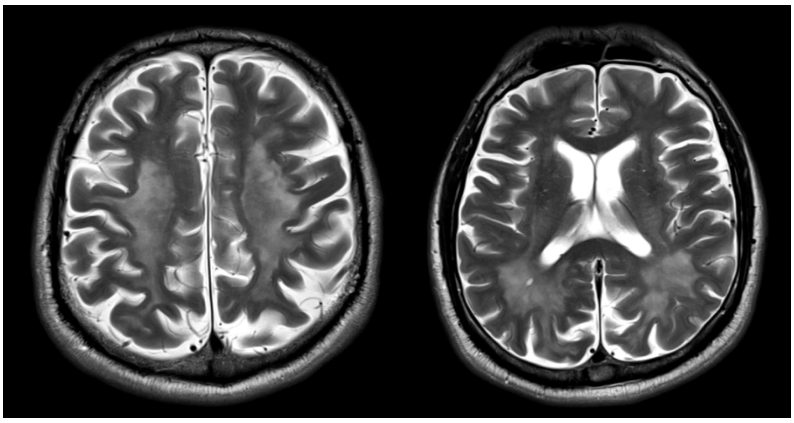

Supplementary Figure 3. Imaging appearance (axial T2-weighted MRI images) of P19 with a homozygous variant in MRPS22

Species		Alignment
Human		202 R M I Q V Y F P K E G R K I L T P I I F K E E
mutated	not conserved	202 R M I Q V Y F P K E G **H** K I L T P I I F K E E
Ptroglodytes	all identical	202 R M I Q V Y F P K E G R K I L T P I I F K E E
Mmulatta	all identical	202 R M I Q I Y F P K E G R K V L T P I I F K E E
Mmusculus	all identical	201 R V I Q I Y F P K E G R R V L P P V I F K D E
Ggallus	all identical	136 R M I Q I F F P K E G R R V I P P V L F K D E
Trubripes	all identical	140 R L I Q V Y F P K Q G R Q L T V P L I F Q E Q
Drerio	all identical	184 R I I Q I Y F P K E G R K V T P P P V F K E E
Dmelanogaster	all identical	175 R M N Q L Y F P L E G R Q S Y T P R M F AL E E
Celegans	all identical	176 R M N R T Y Y Q K P N R S V N P P P L F S D P
Xtropicalis	all identical	141 R M I Q V Y F P H E G R K L V P P P V F Q A E

Conservation of c. 605G>A, p. R202H in *MRPS22*

Supplementary References

Dufresne D, Hamdan FF, Rosenfeld JA, Torchia B, Rosenblatt B, Michaud JL, et al. Homozygous deletion of Tenascin-R in a patient with intellectual disability. J. Med. Genet. 2012; 49: 451–4.

ExAC. No Title [Internet]. [cited 2003 May 20] Available from: http://exac.broadinstitute.org

Fuss B, Wintergerst ES, Bartsch U, Schachner M. Molecular characterization and in situ mRNA localization of the neural recognition molecule J1-160/180: a modular structure similar to tenascin. J. Cell Biol. 1993; 120: 1237–49.

Saada A, Shaag A, Arnon S, Dolfin T, Miller C, Fuchs-Telem D, et al. Antenatal mitochondrial disease caused by mitochondrial ribosomal protein (MRPS22) mutation. J. Med. Genet.

2007; 44: 784–6.

Smits P, Saada A, Wortmann SB, Heister AJ, Brink M, Pfundt R, et al. Mutation in mitochondrial ribosomal protein MRPS22 leads to Cornelia de Lange-like phenotype, brain abnormalities and hypertrophic cardiomyopathy. Eur. J. Hum. Genet. 2011; 19: 394–9.

Wintergerst ES, Fuss B, Bartsch U. Localization of janusin mRNA in the central nervous system of the developing and adult mouse. Eur. J. Neurosci. 1993; 5: 299–310.
