# Supplementary material for: Clinical and genetic characterization of leukoencephalopathies in adults
Source: Brain. 2017 Mar 2;140(5):1204–11. doi: 10.1093/brain/awx045 (PMC5405235; doi:10.1093/brain/awx045)
Supplement: Supplementary Data [file awx045_Supp.zip › brain-2016-01749-File007.pdf]

ID	Clinical Characteristics										MRI findings																
											T1 Signal	Cerebral WM T2 Hyperintensity						Infra-tentorial Involvement			Associated Features						
	Sex	Age at onset	Family History	Cognitive	UMN	Parkinsonism	Ataxia	Neuropathy	Seizures	Additional	WM T1 Signal (↓ hypointense, ↔ isointense)	Confluent (C) or Multifocal (M)	Periventricular	Subcortical U Fibres	Frontoparietal	Temporal	Occipital	Brainstem	Cerebellum	Spinal Cord	DWI abnormality	Thin Corpus Callosum	WM rarefaction	Calcifications	Infarcts	Microhaemorrhages	Contrast enhancement
**N1**	f	40	AD	+	+	-	-	-	-	-	↓	C	+	-	+	-	-	-	-	-	-	-	-	-	-	N/A	N/A
**N2**	f	50	AD	+	+	-	-	-	-	-	↓	C	+	-	+	-	-	-	-	-	-	-	-	-	-	N/A	N/A
**N3**	m	67	No	+	+	-	-	-	-	-	↓	C	+	-	+	-	-	+	-	-	-	-	-	-	+	+	-
**N4**	m	35	No	+	+	-	+	-	-	-	↓	C	+	-	+	-	-	+	-	+	-	+	+	-	-	-	-
**N5**	f	40	No	+	+	+	+	-	-	-	↔	C	+	-	+	-	-	+	-	+	-	-	-	-	-	-	-
**N6**	f	23	AR	-	+	+	+	-	-	-	↓	C	+	-	+	-	-	-	-	-	-	-	-	-	-	-	-
**N7**	m	29	AR	-	+	+	+	-	-	-	↓	C	+	-	+	-	-	-	-	-	-	-	-	-	-	N/A	-
**N8**	f	48	No	+	+	+	+	-	-	-	↓	C	+	-	+	-	-	+	+	-	-	+	-	-	-	-	-
**N9**	m	22	No	+	+	-	-	-	-	-	↓	C	+	-	+	-	-	-	-	-	-	-	-	-	-	-	-
**N10**	m	39	No	+	+	+	+	-	-	Myoclonus	↓	C	+	-	+	-	-	-	-	-	-	-	-	-	-	-	-
**N11**	f	30	No	+	+	-	-	-	-	-	↓	C	+	-	+	-	-	-	-	-	-	+	-	-	-	N/A	N/A
**N12**	m	50	AD	+	+	-	-	-	-	-	↓	C	+	-	+	-	-	-	-	-	-	-	-	-	-	-	-
**N13**	m	34	No	+	-	-	-	-	-	-	↓	C	+	-	+	-	-	-	-	-	-	-	-	-	-	N/A	-
**N14**	f	46	No	+	-	-	-	-	-	-	↓	C	+	-	-	-	-	-	-	-	-	-	-	-	-	-	-
**N15**	f	67	No	+	-	+	-	-	-	-	↓	C	+	-	+	-	-	-	-	-	-	-	-	-	-	N/A	N/A
**N16**	m	20	No	+	+	+	+	-	+	-	↔	C	+	-	+	-	-	-	-	-	-	-	-	-	-	-	-
**N17**	m	50	AD	+	+	-	+	-	-	-	↓	C	+	-	+	-	+	-	+	-	+	-	-	-	-	N/A	-
**N18**	f	35	No	+	+	-	+	+	-	-	↓	C	+	-	+	-	-	-	-	-	-	+	-	-	-	-	-
**N19**	f	52	No	+	+	-	-	-	-	-	↓	C	+	-	+	-	-	-	-	-	-	-	-	-	-	N/A	N/A
**N20**	m	37	No	+	+	-	-	-	-	-	↓	C	+	-	+	-	-	-	-	-	-	-	-	-	-	-	-
**N21**	f	44	No	+	+	-	+	-	-	-	↓	C	+	-	+	-	+	+	+	-	-	-	-	-	-	-	-
**N22**	m	16	No	+	-	-	-	-	+	Optic Atrophy	↓	C	+	-	+	-	-	-	+	-	-	+	-	-	-	-	-
**N23**	f	30	No	-	+	-	-	-	-	-	↓	C	+	-	+	-	-	-	-	+	-	-	-	-	-	N/A	-
**N24**	m	18	No	+	-	-	+	-	-	Myoclonus	↓	C	+	-	+	-	-	-	+	-	-	-	-	-	-	-	-
**N25**	f	34	AD	+	+	+	+	-	-	-	↓	C	+	-	+	-	-	-	-	-	+	-	-	-	-	-	-
**N26**	f	45	No	+	+	-	-	-	-	Myoclonus	↓	C	+	-	+	+	-	-	-	-	-	-	-	-	-	-	-
**N27**	f	53	No	+	+	-	-	-	-	-	↓	C	+	-	+	-	+	+	-	-	-	-	-	-	-	N/A	N/A
**N28**	m	51	AD	+	-	-	+	-	-	-	↓	C	+	-	+	-	-	+	+	-	-	+	-	-	-	-	-
**N29**	m	34	No	+	+	-	-	-	+	-	↓	C	+	-	+	-	-	-	-	-	-	-	-	-	-	-	-
**N30**	f	56	No	+	-	-	-	-	-	-	↓	C	+	-	+	-	-	-	-	-	-	-	-	-	+	N/A	N/A
**N31**	m	53	No	+	-	-	+	-	-	-	↓	C	+	-	+	-	-	-	-	-	-	-	-	-	-	-	-
**N32**	f	20	AD	-	-	-	-	-	-	Severe migraine	↓	C	+	-	+	-	-	-	-	-	-	-	-	-	-	-	-
**N33**	m	18	No	-	+	-	-	-	-	Cardiomyopathy	↓	C	+	-	+	-	-	-	-	-	+	-	+	-	-	-	-
**N34**	f	56	No	+	+	+	-	-	-	-	↓	C	+	-	+	-	-	-	-	-	-	-	-	-	-	-	-

**N35**	m	16	No	+	+	+	+	+	+	-	↓	C	+	-	+	-	+	+	+	-	-	+	+	-	-	-	-
**N36**	f	51	No	+	+	+	+	-	-	-	↓	C	+	-	+	-	-	+	+	-	-	-	-	-	-	N/A	N/A
**N37**	f	41	No	+	+	-	-	-	+	-	↓	C	+	-	+	-	-	-	-	-	-	-	-	-	-		-
**N38**	f	12	No	+	+	-	+	+	-	Premature Ovarian Failure	↓	C	+	-	+	-	-	-	-	-	-	-	-	-	-	-	-
**N39**	m	66	No	+	+	-	+	-	-	Vertical gaze palsy	↓	C	+	-	+	-	-	-	+	-	-	-	-	-	+	+	-
**N40**	m	40	No	+	+	+	-	-	-	-	↓	C	+	-	+	-	-	-	-	-	-	-	-	-	-	N/A	-
**N41**	f	8	No	+	+	-	-	+	-	Myopathy	↓	C	+	-	+	-	-	-	-	-	-	-	-	-	-	-	-
**N42**	m	31	AD	+	+	+	+	-	-	Axonal spheroids and pigmented glia in brain	↓	C	+	-	+	-	-	-	-	-	-	-	-	-	-	-	-
**N43**	m	21	AD	+	-	-	+	-	-	-	↓	C	+	-	+	-	-	-	-	+	-	-	-	-	-	-	-
**N44**	f	8	No	+	-	-	+	-	-	Cardiac Valve Disease	↓	C	-	-	-	-	-	-	-	-	-	-	-	-	-	+	-
**N45**	m	15	No	+	-	-	+	-	+	Adrenal Insufficiency	↓	C	+	-	+	-	-	-	-	-	-	-	-	-	-	-	-
**N46**	m	47	No	+	+	+	+	-	-	-	↓	C	+	-	+	-	-	+	-	-	-	+	-	-	-	N/A	N/A
**N47**	f	8	No	+	-	+	-	-	-	Premature Ovarian Failure	↓	C	+	-	+	-	-	-	-	-	-	+	-	-	-	-	-
**N48**	f	Childhood	AR	+	-	-	-	-	-	Nephrotic Syndrome	↓	C	+	-	-	-	-	-	-	-	-	+	-	-	-	-	-
**N49**	f	Childhood	AR	+	-	-	-	-	-	Nephrotic Syndrome	↓	C	+	-	-	-	-	-	-	-	-	+	-	-	-	-	-
**N50**	m	35	No	+	+	+	+	-	-	-	↓	C	+	-	+	-	-	-	-	-	-	-	-	-	-	-	-
**N51**	f	53	AD	+	+	-	-	-	-	-	↓	C	+	-	+	-	-	-	-	-	-	+	-	-	-	N/A	-
**N52**	m	55	No	+	-	-	+	-	-	Autonomic Features	↓	C	+	-	+	-	+	+	+	-	-	-	-	-	-	-	-
**N53**	m	44	No	+	-	+	+	-	-	-	↓	C	+	-	+	-	+	-	-	-	-	-	-	-	-	N/A	-
**N54**	m	37	No	-	+	-	-	-	-	-	↓	C	-	-	+	-	-	-	+	-	-	-	-	-	-	-	-
**N55**	m	18	No	-	+	-	-	-	-	-	↓	C	+	-	+	-	-	-	-	-	-	-	-	-	-	-	-
**N56**	m	40	No	+	+	+	-	-	-	-	↓	C	+	-	+	-	-	-	-	-	-	-	-	-	-	N/A	-
**N57**	f	35	No	+	+	-	-	-	-	-	↓	C	+	-	+	-	-	-	-	-	-	-	-	-	-	-	-
**N58**	f	50	AD	-	-	-	-	-	-	Severe migraine	↓	C	+	-	+	-	-	+	-	-	-	-	-	-	-	N/A	N/A
**N59**	m	52	No	+	-	-	-	-	-	-	↓	C	+	-	+	-	-	-	-	-	-	-	-	-	-	-	-
**N60**	f	51	No	+	-	-	-	-	-	-	↓	C	+	-	+	-	-	-	-	-	-	-	-	-	-	-	-
**N61**	f	33	No	+	-	-	-	-	+	-	↓	C	+	-	+	-	-	-	-	-	-	-	-	-	-	-	-
**N62**	f	30	No	+	+	-	+	-	-	-	↓	C	+	+	+	-	-	-	-	-	-	-	-	-	-	-	-
**N63**	f	Childhood	No	+	+	-	+	+	+	-	↓	C	+	-	+	-	-	+	-	-	-	-	-	-	-	-	-
**N64**	m	35	No	-	-	-	-	-	-	-	↓	C	+	-	+	-	-	-	-	-	-	-	-	-	-	-	-
**N65**	f	60	AD	+	-	-	-	-	-	-	↓	C	+	-	+	-	-	-	-	-	-	-	-	-	+	N/A	N/A
**N66**	f	72	No	+	+	+	+	-	-	-	↓	C	+	-	+	-	-	-	-	-	-	-	-	-		-	-
**N67**	m	20	No	-	+	-	-	-	-	-	↓	C	+	-	+	+	-	-	-	-	-	-	-	-	-		-
**N68**	m	15	No	+	+	-	+	-	-	-	↓	C	+	-	+	-	-	+	+	-	-	+	-	-	-	-	-
**N69**	f	15	No	+	+	+	+	+	-	-	↓	C	+	-	+	-	-	-	-	-	-	-	-	-	-	-	-
**N70**	m	8	AD	+	+	+	+	-	-	-	↓	C	+	-	+	-	-	-	-	-	+	+	-	-	-	-	-
**N71**	f	63	No	-	+	-	-	-	-	-	↓	C	+	-	+	-	-	-	-	-	+	-	-	-	+	N/A	N/A

(Legend: +present, -absent, N/A information not available, AD autosomal dominant, AR autosomal recessive)
